# Feline Panleukopenia Outbreaks and Risk Factors in Cats in Animal Shelters

**DOI:** 10.3390/v14061248

**Published:** 2022-06-08

**Authors:** Teresa Rehme, Katrin Hartmann, Uwe Truyen, Yury Zablotski, Michèle Bergmann

**Affiliations:** 1 Clinic of Small Animal Medicine, Centre for Clinical Veterinary Medicine, LMU Munich, Veterinaerstrasse 13, 80539 Munich, Germany; hartmann@lmu.de (K.H.); y.zablotski@med.vetmed.uni-muenchen.de (Y.Z.); n.bergmann@medizinische-kleintierklinik.de (M.B.); 2 Institute of Animal Hygiene and Veterinary Public Health, Veterinary Faculty, University of Leipzig, An den Tierkliniken 1, 04103 Leipzig, Germany; truyen@vetmed.uni-leipzig.de

**Keywords:** feline panleukopenia virus, FPV, shedding, shelter management, shelter medicine, vaccination, vaccine virus, canine parvovirus, CPV

## Abstract

(1) Background: This study aimed to determine the risk factors for outbreaks of feline panleukopenia in shelters. (2) Methods: Four shelters (A−D) with 150 cats were included. Fecal samples were analyzed by parvovirus real-time polymerase chain reaction (qPCR), including culture and sequencing of qPCR-positive samples. Information on cats, husbandry, hygiene, and infection management was evaluated to determine risk factors for feline panleukopenia and parvovirus shedding by logistic regression. (3) Results: Feline panleukopenia occurred in 28.0% (42/150) of cats (0 in shelter D). Shedding was found in 48.7% (73/150) (A: 21/73; B: 29/73; C: 7/73; D: 16/73). Of 73 qPCR-positive fecal samples, 65.8% (48/73) were culture-positive; sequencing revealed feline panleukopenia virus (FPV) isolates in 34/48 samples and vaccine virus isolate in 14/48; canine parvovirus was not detected. Presence of feline panleukopenia was significantly more likely in cats from shelter A (*p* < 0.05), unvaccinated cats (*p* < 0.001), and young cats (4 weeks to 2 years; *p* = 0.008). Parvovirus shedding was significantly more common in young cats (*p* < 0.001), cats with feline panleukopenia (*p* = 0.033), and group-housed cats (*p* = 0.025). (4) Conclusions: Vaccination is the most important measure to reduce the risk of feline panleukopenia in shelters. Risk of parvovirus shedding is especially high in young, group-housed cats.

## 1. Introduction

Feline panleukopenia is a highly contagious disease of cats, often with fatal outcome [1,2]. Feline panleukopenia virus (FPV) is a non-enveloped, icosahedral, single-stranded DNA virus [3,4,5] that belongs to the genus Protoparvovirus [6,7]. It is very resistant to physical factors and chemical substances and can remain infectious in the environment for months to years [8,9,10,11]. This is why the virus is not only transmitted through direct contact, but also indirectly through contaminated individuals or not properly disinfected items. In shelters, staff members and equipment can act as carriers and therefore pose a risk for unprotected cats [3,12]. In addition, healthy immunocompetent cats with asymptomatic infection can shed infectious FPV [13] and canine parvovirus (CPV) [14] and thus be an important source for contamination of the environment [13]. 

Due to its single-stranded DNA, FPV requires cellular DNA polymerase for replication and therefore rapidly dividing cells in the S-phase of division [3,4]. Bone marrow, lymphatic tissue, and intestinal epithelial cells are particularly affected [3,5,15,16,17]. The most common clinical signs are fever, vomiting, diarrhea, anorexia, and/or dehydration [1,3,5,16,17]. Especially in kittens, the course of disease is often peracute, which leads to death [1,18].

Feline panleukopenia virus has become less prevalent in the domestic cat population over the last decades due to widespread vaccination [3,12,15]. Outbreaks in shelter cats are, however, still commonly reported and often related to a high number of fatalities [1,12,15,19,20]; for example, at least 350 fatalities have been recorded during 3 recent outbreaks in shelters in Australia [1,21], concluding that dealing with feline panleukopenia outbreaks poses a great challenge especially in animal shelters. Besides vaccination management, hygiene, disinfection, and quarantine measures are considered to play an important role to avoid these outbreaks [12,17,22]. In shelters, it is recommended to use modified live virus vaccines (MLV) because of their rapid onset of immunity [3]. In case of feline panleukopenia outbreaks, passive immunization can be performed by using commercial hyperimmune serum containing anti-FPV antibodies [22].

The present study investigated feline panleukopenia outbreaks in animal shelters. The aim was to describe disease outbreaks and risk factors in these shelters and compare the data to those of a shelter without recent history of feline panleukopenia. 

## 2. Materials and Methods

### 2.1. Shelters and Cats Population

In total, 4 animal shelters in Bavaria, Germany, were included and sampled between June and December 2020. Three of the 4 shelters (animal shelter A–C) reported feline panleukopenia outbreaks at the time of sampling. An outbreak was defined as a sudden increase in frequency of feline panleukopenia cases, related to time, place, and observed population [23]. The fourth shelter (animal shelter D) had no history of feline panleukopenia within the last 24 months and was included for comparative reasons. The protocol of the present study was accepted by the ethical committee of the Centre for Clinical Veterinary Medicine of the LMU Munich, Germany (reference number 230-04-08-2020).

Collected data from the shelters included background information of each cat (signalment: breed, gender, age group according to the feline life stage guidelines [24], and neutering status; housing conditions: animal shelter origin and group-housing; history and presence of clinical signs of feline panleukopenia as well as methods and results of direct FPV detection; immunization status: active and/or passive immunization and correct vaccination series according to current vaccination guidelines [25,26]), as well as general information about the shelters’ management (vaccination management; hygiene management: handling and measures for new incoming and/or ill cats, disinfection, regular cleaning/disinfection, cleaning of litterboxes, cages and other equipment). 

Fecal (*n* = 88) and sera (*n* = 138) samples of the shelter cats were collected. Of the 88 fecal samples, 47 samples were assignable to individual cats, and 41 samples were mixed fecal samples from cats that were kept in groups (of 2 to 5 cats). Blood sampling of 12 cats was not possible due to aggressiveness.

Fecal samples were analyzed for parvovirus DNA by real-time polymerase chain reaction (qPCR). Parvovirus from qPCR-positive samples was isolated in cell culture and submitted to VP2 gene sequencing. Results of the not-assignable 41 mixed fecal samples were subsequently applied for all cats that were housed in the same group. Serum samples were analyzed by serum neutralization test (SNT) for anti-FPV antibodies. 

Diagnosis of feline panleukopenia was based on the presence of clinical signs (acute onset of anorexia, diarrhea, vomiting, and/or fever [1,3,12,16]) observed by shelters’ technicians and/or veterinarians; in all cats with clinical suspicion, feline panleukopenia was confirmed by direct virus detection (using point-of-care (POC) antigen test and/or PCR in feces [27,28,29]).

Overall, 150 cats from the 4 shelters were included (shelter A: *n* = 47; shelter B: *n* = 48; shelter C: *n* = 21; shelter D: *n* = 34). Intake of new cats was not stopped in the affected shelters. After the beginning of the outbreaks, 5 cats were newly admitted to shelter A, 23 to shelter B, and 7 to shelter C.

In total, 97.3% (146/150) of the cats were domestic shorthair (DSH); the other cats belonged to different breeds (European Longhair (*n* = 1), Maine Coon (*n* = 1), Persian (*n* = 1), and Exotic Shorthair (*n* = 1)); 49.3% (74/150) of the cats were female and 50.7% (76/150) were male. Age ranged from 4 weeks (w) to 15 years (y) and median age was 122 days (≈4 months); 80.0% (120/150) of the cats were assigned to the age group “young cats” (≤2 y), according to feline life stage guidelines [24]. In total, 71.3% (107/150) of the cats were kept in groups of 2 to 5 cats. Overall, 17.3% (26/150) of the cats had not received prior immunization against FPV. Of 82.7% (124/150) of cats with prior immunization, 62.9% (78/124; shelter A: 10/47; shelter B: 33/48; shelter C: 10/21; shelter D: 25/34) were vaccinated with MLV vaccines from 4 different manufacturers (Boehringer Ingelheim, Lyon, France; MSD Tiergesundheit, Unterschleißheim, Germany; Virbac, Carros, France; Zoetis, New Jersey, America). The remaining 38.7% (48/124; shelter A: 22/47; shelter B: 10/48; shelter C: 10/21; shelter D: 6/34) had previously received a commercial hyperimmune serum of equine origin containing anti-FPV antibodies (Feliserin PLUS^®^, Selectavet, Weyarn, Germany) according to the manufacturer’s instructions. Two cats had received both, vaccination and hyperimmune serum. Overall, 62.5% (30/48) of the cats received hyperimmune serum and had feline panleukopenia; 60.4% (29/48) of the cats received hyperimmune serum and had fecal parvovirus shedding; and 47.9% (23/48) of the cats received hyperimmune serum, had feline panleukopenia and simultaneously had fecal parvovirus shedding.

### 2.2. Real-Time Polymerase Chain Reaction to Detect Viral DNA

Viral DNA was extracted from 200 milligrams (mg) feces using the QIAamp DNA Stool Mini Kit (Qiagen, Venlo, The Netherlands), as recommended by the manufacturer. Twenty-four samples were processed at the same time, including 1 extraction control phosphate-buffered saline (PBS). Presence of FPV DNA was determined by qPCR targeting a 201 bp region of the VP2 gene, as described previously [13]. Real-time PCR was performed using the PCR-BIO Probe Mix No-ROX (PCR Biosystems, London, England) on a Stratagene Mx3000P real-time cycler. A 20 microliter (µL) reaction was set up containing 10.0 µL 2 × PCRBIO Probe Mix No-ROX, 5.20 µL of PCR water, 0.4 micromolar (µM) of forward primer 5’-TGG AAC TAG TGG CAC ACC AA-3’, 0.3 µM of reverse primer 5’-AAA TGG TGG TAA GCC CAA TG-3’, 0.2 µM of probe 6FAM-CAGGTGATGAATTTGCTACAGG-BBQ, and 3 µL of extracted DNA. The cycling parameters were 95 °C for 3 min, followed by 40 cycles 95 °C for 10 s and 60 °C for 25 s. The limit of detection (95% probability (P)) was 77 copies/reaction (P = 0.95, standard error = 13.86) using the plasmid-derived DNA standard. In each qPCR run, the extraction controls and a no-template control (negative control, PCR water) were included. All diagnostic steps (e.g., DNA extraction, preparation of PCR master mix, qPCR and clean-up of amplification products) were conducted in separate rooms (one-way principle) to avoid cross-contamination of samples.

### 2.3. Virus Culture to Identify Replicating Virus

For all qPCR-positive fecal samples, virus culture was performed. Two-hundred milligrams of feces were suspended in 2.0 milliliters (mL) PBS (pH 7.2), centrifuged at 3000× *g* for 5 min, and the supernatant was filtered through a 0.22 micrometer (µm) syringe filter. Afterward, 100 µL of these filter suspensions were used to inoculate Crandell Rees feline kidney (CRFK) cells maintained in Dulbecco’s medium (Biowest, Nuaillé, France) supplemented with 5% fetal calf serum (Sigma Aldrich, St. Louis, MO, USA), 1% non-essential amino acids (Gibco™ by Thermo Fisher Scientific, Waltham, MA, USA) and 1% penicillin-streptomycin (Gibco™ by Thermo Fisher Scientific, Waltham, MA, USA). Cultures were incubated at 37 °C, 5% CO_2_. On day 7 after incubation, each culture was subcultured, and growth of virus in subculture was determined by qPCR as described above.

### 2.4. VP2 Gene Sequence Analysis to Differentiate between FPV and Vaccine Virus Isolates and CPV

All qPCR-positive fecal samples were submitted to VP2 gene sequencing using primers M1 and M2 as described previously with a modified touchdown protocol [30]. A 25 µL reaction was set up containing 12.5 µL Thermo Scientific™ DreamTaq PCR Master Mix (2X) (Thermo Fisher Scientific, Waltham, MA, USA), 8.5 µL of PCR water, 0.4 µM of forward and reverse primers, and finally, 2.0 µL of sample DNA was added. The cycling parameters were 95 °C for 2 min, followed by 35 cycles 95 °C for 30 s, 60 °C for 30 s and again 72 °C for 60 s. The final extension was 72 °C for 10 min. Clean-up of amplification products was performed using the NucleoSpin Gel and PCR Clean-up kit (Macherey-Nagel, Düren, Germany) following the manufacturer’s instructions. Direct sequencing of qPCR products was carried out by Eurofins Genomics. Determination of open reading frames (ORFs) and subsequent amino acid alignments, with corresponding sequences retrieved from the GenBank database was performed. Furthermore, results of the VP2 gene sequence analysis were compared with the sequence of an FPV vaccine virus isolate (PLI IV) contained in the vaccine Purevax RCP^®^ (Boehringer Ingelheim, Lyon, France) [13] to differentiate between FPV isolates and vaccine virus isolate. This vaccine strain contains an amino acid mutation (isoleucine) in VP2 in position 101.

### 2.5. Serum Neutralization Test to Detect Anti-Parvovirus Antibodies

Dulbecco’s MEM (Biowest, Nuaillé, France)-maintained CRFK cells were supplemented with 5% fetal calf serum (Sigma Aldrich, St. Louis, MO, USA), 1% nonessential amino acids (Gibco™ by Thermo Fisher Scientific, Waltham, MA, USA) and 1% Penicillin–Streptomycin (Gibco™ by Thermo Fisher Scientific, Waltham, MA, USA) at 37 °C, 5% CO_2_. The cats’ sera were then heat-inactivated at 56 °C for 30 min. A 96-well microtiter plate was prepared with 60 µL PBS (pH 7.2) in each well. As the next step, the first row was inoculated with 60 µL of each cat-serum and afterward serially diluted at steps of 1:2 in every following row. Each dilution was mixed with 60 µL of the FPV Virus 292 (100 TCID_50_) and incubated at 37 °C for 60 min. With 100 μL of these serum/virus mixtures, the CRFK cells seeded were inoculated. The plates were incubated for 5 days at 37 °C, 5% CO_2_. Thereafter, cells were fixed using acetone (>99.9%)/methanol (>99.9%) 1:1 (vol/vol) at −20 °C for 20 min. For virus staining, a mixture of parvovirus-specific monoclonal antibodies (kindly provided by Colin Parrish [31]) was applied and incubated overnight at 37 °C. The next day, a fluorescein isothiocyanate-conjugated goat anti-mouse IgG (H+L) conjugate (Dianova, Hamburg, Germany) was applied and incubated over night at 37 °C. Finally, samples were analyzed using a Leica DMIL fluorescence microscope. A FPV strain from the Institute of Animal Hygiene and Veterinary Public Health, University of Leipzig, was used as a positive control. A titer < 10 was considered negative, titers ≥ 10 were regarded as positive. All samples were run in duplicates in the same batch.

### 2.6. Statistical Analysis

Statistical analysis was performed using R Version 3.4.4. Risk factors for (1) “presence of feline panleukopenia” and (2) “fecal parvovirus shedding” were determined in relation to the cats’ animal shelter (origin) by univariate Bayesian logistic regression. In addition, influence of “hygiene/husbandry factors” and “medical factors” was evaluated in relation to presence of feline panleukopenia and to fecal parvovirus shedding by Bayesian logistic regression [32]. Factors that proved to be significant in relation to presence of feline panleukopenia and factors that proved to be significant in relation to fecal parvovirus shedding in univariate analysis (*p* value (*p*) ≤ 0.05) became part of the multivariate model, in which they were examined for multicollinearity via variance inflation factor (VIF) [33]. Variables with a VIF ≥ 5 suggested multicollinearity and were excluded from further analyses. Variables with a VIF < 5 were considered as not being multicollinear and subsequently used for further stepwise backward elimination analysis based on the Akaike information criterion (AIC) [34]. Odds ratios (OR) with 95% confidence intervals (CI) and *p* values were used to show the strength of the relationship between risk factors and response variables (presence of feline panleukopenia and fecal parvovirus shedding). 

## 3. Results

### 3.1. Presence of Feline Panleukopenia

At the time of sampling, feline panleukopenia occurred in 28.0% (42/150; 95% CI: 21.4–35.7%) of all cats (shelter A 59.6% (29/47; 95% CI: 45.4–72.3%); shelter B 17.5% (8/48; 95% CI: 9.2–30.6%); shelter C 25.4% (5/21; 95% CI: 11.6–47.0%); shelter D 0% (0/34; 95% CI: 0–19.4%)) (Table 1). Most of the cats with feline panleukopenia (95.2%; 40/42) were young cats (≤2 y) with a mean age of 77 days (≈2.5 months; range: 60–487 days) and were housed in groups (76.2%; 32/42) (Table 2). In total, 23.8% (10/42) of the cats with feline panleukopenia had no prior immunization; 4.8% (2/42) of the cats with feline panleukopenia were vaccinated with MLV vaccines prior to onset of clinical signs; and 71.4% (30/42) had received hyperimmune serum. 

Overall, the 3 outbreak-affected shelters (shelter A, B, and C) recorded 76 feline panleukopenia fatalities during these outbreaks (shelter A: *n* = 3; shelter B: *n* = 62; shelter C: *n* = 11; shelter D: *n* = 0).

### 3.2. Fecal Parvovirus Shedding

Parvoviral DNA shedding was detected in 48.7% (73/150; 95% CI: 40.8–56.6%) of the cats (shelter A 44.8% (21/47; 95% CI: 31.8–58.6%); shelter B 60.0% (29/48; 95% CI: 45.9–72.7%); shelter C 34.0% (7/21; 95% CI: 17.6–55.3%); shelter D 47.0% (16/34; 95% CI: 31.4–63.2%)) (Table 1).

Fecal samples of 65.8% (48/73) of the qPCR-positive cats were also positive in virus culture; in 70.8% (34/48) of these fecal samples, DNA of FPV isolates, and in 29.2% (14/48), DNA of vaccine virus (VV) isolates was detected (shelter A: 21/21 FPV isolate; shelter B: 13/18 FPV isolate, 5/18 VV isolate; shelter C: no sequencing possible; shelter D: 9/9 VV isolate). Of the cats that shed vaccine virus isolates, 92.9% (13/14) had been actively vaccinated within the last 4 to 25 days (median 5 days). DNA from FPV isolates was detected in feces from 5 cats that had been vaccinated with a vaccine from another manufacturer, of which vaccine virus isolate was unknown, within the last 23 days. DNA from vaccine virus isolate was detected in 1 cat, although this cat had not been vaccinated since entering the shelter (4 days before sampling). Canine parvovirus (CPV) DNA was not detected in any of the fecal samples. 

Signs of feline panleukopenia and simultaneous shedding of FPV were present in 39.7% (29/73) of the shedding cats; 31.0% (13/42) of the cats with feline panleukopenia did not shed viral DNA. Conversely, 60.3% (44/73) of the shedding cats did not have feline panleukopenia and were considered clinically healthy (Table 3).

### 3.3. Anti-Parvovirus Antibodies

Overall, parvovirus antibodies were detected in 87.7% (121/138) of the shelter cats (median titer: 1.280; range: 10–10.240); 55.4% (67/121) of these cats had been vaccinated with MLV vaccines previously and 33.1% (40/121) had received hyperimmune serum containing anti-FPV antibodies (2 cats had received both, vaccination and hyperimmune serum). Feline panleukopenia was observed in 76.9% (30/39) (Table 2) and fecal parvovirus shedding in 82.6% (57/69) of the cats with anti-FPV antibodies (Table 3).

### 3.4. Risk Factors for Feline Panleukopenia

**Figure 1:**
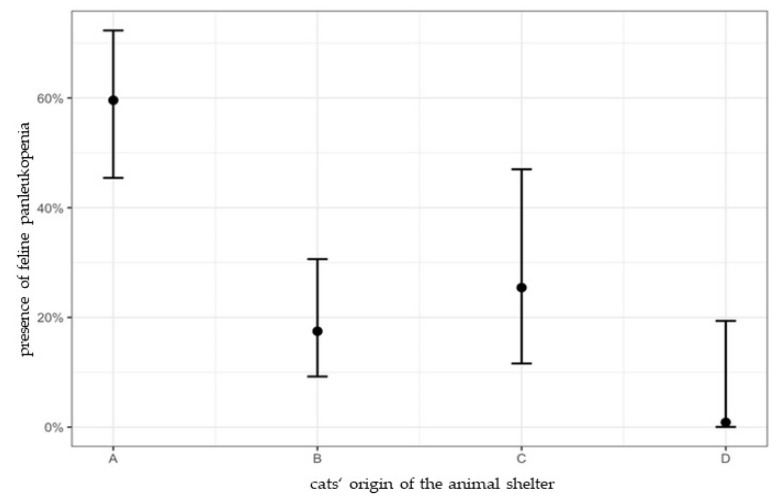
Percentage of cats with feline panleukopenia in shelter A, B, C, and D with 95% confidence intervals. In shelter A, 59.6% (95% CI: 45.4–72.3%) of cats, in shelter B, 17.5% (95% CI: 9.2–30.6%) of cats, in shelter C, 25.4% (95% CI: 11.6–47.0%) of cats, and in shelter D, 0% (95% CI: 0–19.4%) of cats had feline panleukopenia. Univariate Bayesian logistic regression revealed that cats from shelter A had a significantly higher risk for feline panleukopenia in comparison to cats from shelter B, C, and D (A/B: *p* < 0.001, OR: 6.96; A/C: *p* = 0.044, OR: 4.32; A/D: *p* = 0.014, OR: 167.46). Animal shelter D had no history of feline panleukopenia within the last 24 months and was included for comparative reasons.

The cats’ origin had a significant influence on presence of feline panleukopenia. Cats from shelter A had a significantly higher risk for feline panleukopenia in comparison to cats from shelter B, C, and D (A/B: *p* < 0.001, OR: 6.96; A/C: *p* = 0.044, OR: 4.32; A/D: *p* = 0.014, OR: 167.46). Risk for feline panleukopenia for cats from shelter B, C, and D did not differ significantly (B/D: *p* = 0.254; C/B: *p* = 0.907; C/D: *p* = 0.127) (Figure 1).

In univariate analysis, 5 individual risk factors (age group, FPV antibody titer, application of hyperimmune serum, vaccination, correct immunization series) were significantly associated with feline panleukopenia (Table 2); examination on multicollinearity showed low correlation (VIF < 5) and thus, all 5 factors were included in multivariate analysis. Young age (≤2 y) (*p* = 0.008; OR: 71.8; 95% CI: 2.91–1772.7) and lack of vaccination (*p* < 0.001; OR: 46.49; 95% CI: 11.69–184.91) were the factors that proved to be significantly associated with feline panleukopenia (Table 2). Of the husbandry and hygiene factors, 11 were significantly associated in univariate analysis with presence of feline panleukopenia (Table 2); all of these factors were highly correlated (VIF ≥ 5), and thus multivariate analysis was not performed.

### 3.5. Risk Factors for Fecal Parvovirus Shedding

**Figure 2:**
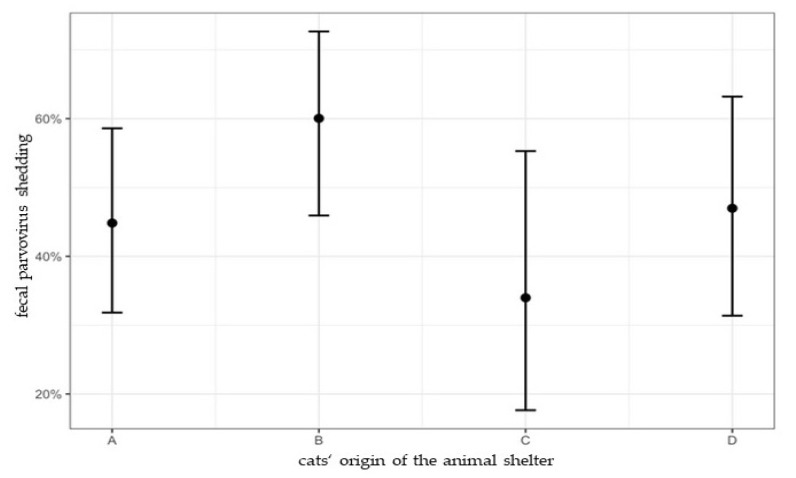
Percentage of cats with fecal parvovirus shedding in shelter A, B, C, and D with 95% confidence intervals. In shelter A, 44.8% (95% CI: 31.8–58.6%) of cats, in shelter B, 60.0% (95% CI: 45.9–72.7%) of cats, in shelter C, 34.0% (95% CI: 17.6–55.3%) of cats, and in shelter D, 47.0% (95% CI: 31.4–63.2%) of cats had fecal parvovirus shedding. Fecal parvovirus shedding was detected in shelter D, although no cases of feline panleukopenia were observed in this shelter. Sequencing revealed fecal shedding of vaccine virus in shelter D (Table 1). Univariate Bayesian logistic regression revealed that fecal parvovirus shedding was not significantly associated with the shelter from which the cats originated (B/A: *p* = 0.497; B/C: *p* = 0.220; B/D: *p* = 0.152).

Fecal parvovirus shedding was not significantly associated with the shelter from which the cats originated (B/A: *p* = 0.497; B/C: *p* = 0.220; B/D: *p* = 0.152) (Figure 2). Four individual factors (age group, FPV antibody titer, application of hyperimmune serum, feline panleukopenia) were significantly associated with fecal parvovirus shedding in univariate analysis (Table 3); examination on multicollinearity showed low correlation (VIF < 5) and all factors were included in multivariate analysis. Cats with feline panleukopenia (*p* = 0.033; OR: 2.31; 95% CI: 1.06–5.01) and a young age (≤2 y) (*p* < 0.001; OR: 10.91; 95% CI: 2.82–42.17) were significantly more likely to shed parvovirus (Table 3). Eight husbandry and hygiene factors were significantly associated with fecal parvovirus shedding in univariate analysis (Table 3); all these factors showed low correlation (VIF < 5) and were included in multivariate analysis. Only the factor group-housing proved to be significant; cats that were housed in groups had a higher risk for parvovirus shedding (*p* = 0.025, OR: 2.40, 95% CI: 1.16–5.64) than cats that were housed had a higher risk for parvovirus shedding (*p* = 0.025, OR: 2.40, 95% CI: 1.16–5.64) than cats that were housed alone (Table 3).

## 4. Discussion

The present study compared hygiene, husbandry, and infection management of shelters with and without feline panleukopenia outbreaks and investigated the risk factors for presence of feline panleukopenia and fecal parvovirus shedding. This knowledge is important for minimizing the risk for feline panleukopenia and for optimizing management during outbreak situations in shelter cats. 

Risk factors for feline panleukopenia were the cats’ vaccination status and the cats’ age. Cats that had not been vaccinated against FPV were 47 times more likely to develop feline panleukopenia than vaccinated cats. The comparatively low number of vaccinated cats (21%) in shelter A is likely the most important reason for the higher risk for feline panleukopenia in this shelter. A strict vaccination management is known as being crucial for feline panleukopenia eradication [1,5,12,17,25,26]. Vaccinated cats develop high antibody titers [35,36] that correlate excellently with protection against feline panleukopenia [37]. It is recommended to start vaccination in high risk areas at 4 to 6 weeks of age (low risk areas at 8 weeks), repeating vaccination every 2 to 4 weeks until 20 weeks of age [25,26]. Nevertheless, immediate protection cannot be achieved by active vaccination, especially in kittens, and this is why the outbreak-affected shelters tried to protect incoming susceptible cats by application of a commercial hyperimmune serum. However, multivariate analysis showed that application of hyperimmune serum did not belong to the factors that were significantly associated with the presence of feline panleukopenia, presumably because it was administered too late, when cats were already in an early course of infection. “Age” proved to be another risk factor for feline panleukopenia. Cats ≤ 2 years were 72 times more likely to develop signs of feline panleukopenia than cats > 2 years. The most likely reason is the susceptibility of kittens to feline panleukopenia when maternally derived antibodies (MDAs) decline below protective titers but can still neutralize vaccine antigen [18]. Maternally derived antibodies wane below a concentration that can cause vaccine interference in most of the kittens by 8 to 12 weeks of age [1,25]. In some cases, however, studies showed that MDAs can persist until 16 up to 20 weeks of age [18,38]. Another explanation is that (repeated) exposure to field virus with increasing age boosters immunity [39]. 

Furthermore, various husbandry and hygiene factors were associated with feline panleukopenia in univariate analysis, but all of these factors showed high correlation (VIF ≥ 5), and this is why the exact influence of the factors themselves could not be determined; it is, however, possible that the factors interactively contribute to FPV eradication. A strict hygiene management, including thorough disinfection against non-enveloped viruses [1,22,40], isolation facilities for infected animals, and protective clothing [1,12,22,41] should be available during outbreaks; these procedures might or might not be sufficiently implemented in the affected shelters in the present study, e.g., due to excessive demand on staff members during the outbreak situation, low number of staff members in relation to the number of infected cats, or untrained staff. Another reason that certainly has contributed substantially to the ongoing outbreak situations is the constant intake of new cats, which was allowed in all 3 affected shelters. Intake of new cats was especially common in shelter B, where 23 cats were newly admitted during the ongoing outbreak. This fact, together with the other risk factors, such as lack of vaccination and hygiene management, might have played an important role in the high number of fatalities in shelter B. During outbreaks, however, it is most important (1) to stop the intake of new cats with unknown immune status, or if not feasible, (2) apply hyperimmune serum to susceptible cats before intake (e.g., at veterinary clinics or foster homes) and not after intake in affected shelters (as performed by the shelters in the present study) [12,22,41].

In the present study, feline panleukopenia outbreaks were documented in 3 different animal shelters during early summer until autumn in 2020. An increasing seasonal incidence of feline panleukopenia is well known [1,15,42] and is likely due to the high number of newborn kittens that are especially vulnerable for infection when MDAs wane [18]. Nevertheless, shelter D that was located in the same area as shelters A-C did not report a single case of feline panleukopenia during the same period. 

Fecal parvovirus shedding was found in all four shelters, with an overall prevalence of 48.7% (73/150). The majority of the shedding cats originated from the outbreak-affected shelters A-C (78.1%; 57/73). A high shedding prevalence during feline panleukopenia outbreaks is common and was reported to be even higher in the past (up to 95.5%) [43]. Nevertheless, in the present study, there was no significant association between fecal parvovirus shedding and the shelter origin, likely because a comparatively high number of cats that shed the vaccine virus isolate in shelter D (56.3%; 9/16) were vaccinated with MLV vaccines within the last 4–25 days before sampling; fecal vaccine virus shedding has been demonstrated up to 28 days after FPV vaccination in healthy, adult cats [13]. Besides the vaccine virus isolate, shedding of other FPV isolates was also found in cats without feline panleukopenia (23.5% 8/34) in the present study. Subclinical field virus shedding has been described in shelter-housed cats before, and the prevalence varied from 4% in Italy [43], to 10% in Australia [43], and 37% in the UK [14]; in these previous studies, FPV as well as CPV-2a, -2b, -2c shedding was commonly observed, which led to the assumption that healthy, subclinically shedding cats can be a possible reservoir for other cats and even for dogs [14]. Sources for CPV infections in cats are contact with contaminated dog feces and/or CPV-2-contaminated fomites [3,44]. Although all shelters in the present study were mixed canine and feline shelters, only fecal shedding of FPV could be detected, and none of the cats shed CPV or any new parvovirus variants. Although a previous study from the same region detected fecal shedding of CPV in a client-owned, healthy outdoor cat that came from the same area as the cats from the present study [13], the results suggest that shelter-housed cats do not play an important role as reservoir for CPV-2a, -2b, -2c.

The factor “age” proved to be significantly associated with fecal shedding of FPV, and cats ≤ 2 years were 11 times more likely to shed parvovirus than older ones. FPV infection in general leads to fecal virus shedding from day 2–5 after infection that can last at least 6 weeks, and young cats are highly susceptible to FPV infection [1,3,16,17,45]. During weaning, the mitotic index of the intestinal enterocytes increases due to changes in the kittens’ bacterial gastrointestinal flora, and this leads to a higher parvovirus replication rate and subsequent shedding [46]. Further results of the present study suggest that group-housed cats have a higher risk for fecal shedding of parvovirus. This, however, must be interpreted with caution since not all of the fecal samples from group-housed cats could be individually assigned to one single cat. Subsequently, the results of these mixed fecal samples were applied for all cats that were kept in the same group. However, from an infectiological point of view, fecal transmission of parvovirus in group-housed cats that share litterboxes is very likely since infected cats shed high virus loads (up to over 10^9^ viral particles per gram of feces) [8,9,10,11]. In order to improve feline panleukopenia outbreak management and to prevent fecal transmission, it would be helpful to identify shedding cats by fecal diagnostic tests (e.g., via POC tests) before they are grouped together with other susceptible cats.

In the present study, antibodies against FPV were detected in the majority of the shelter cats (87.7%; 121/138). About one third (33.1%; 40/121) of cats with anti-FPV antibodies were immunized with hyperimmune serum within the last 7 weeks before sampling. In contrast to other studies [13,47,48], anti-FPV antibodies were neither associated with absence (or presence) of feline panleukopenia nor with fecal parvovirus shedding. One explanation for this result is that most of the cats with antibodies and feline panleukopenia or fecal parvovirus shedding had received antibodies via hyperimmune serum containing anti-FPV antibodies; nevertheless, hyperimmune serum was either not effective or was potentially applied too late (already during the incubation period), or at a too low or too short dose and frequency, as it obviously did not prevent development of clinical signs. The highly concentrated anti-FPV antibody serum is usually used as a prophylactic drug in endemic areas for immediate onset of protection lasting over a period of approximately 2–4 weeks [1,3,49]. Nevertheless, in the present study, about half (47.9%; 23/48) of the cats that had received hyperimmune serum developed feline panleukopenia and shed FPV. A lack of beneficial effects has also been reported in a placebo-controlled study in dogs with canine parvovirosis [50].

The main limitation of the present study is that in some of the group-housed cats, fecal samples were not always assignable to individual cats; this was not feasible for the staff members due to the extraordinary outbreak situation. Another limitation was that only 1 shelter without feline panleukopenia outbreak was included. For future studies, more unaffected shelters should be evaluated.

## 5. Conclusions

Consequent vaccination management, especially in endemic environments such as animal shelters, seems to have the most important influence, even more than husbandry and hygiene management, to avoid panleukopenia outbreaks. There is a high risk for feline panleukopenia in young, unvaccinated cats and a high risk for FPV fecal shedding in young, group-housed cats. Therefore, group-housing should not be performed in not yet fully vaccinated cats.

## Figures and Tables

**Table 1:** Percentage of cats with feline panleukopenia and fecal parvovirus shedding (including sequencing results to differentiate between FPV isolates and vaccine virus isolate) in all 4 shelters.

	Shelter A (*n* ^1^ = 47)		Shelter B (*n* = 48)		Shelter C (*n* = 21)		Shelter D (*n* = 34)	
**cats with feline** **panleukopenia**	29/47 (59.6%)		8/48 (17.5%)		5/21 (25.4%)		0/34 (0%)	
**cats with fecal** **parvovirus** **shedding**	21/47 (44.8%)		29/48 (60.0%)		7/21 (34.0%)		16/34 (47.0%)	
	21/21 FPV isolate	0/21 VV ^2^ isolate	13/18 FPV isolate	5/18 VV isolate	NA ^3^	NA	0/0 FPV isolate	9/9 VV isolate

^1^*n*, total number of cats; ^2^ VV, vaccine virus; ^3^ NA, not applicable since VP2 sequencing was not possible for the samples from shelter C.

**Table 2:** Medical, husbandry, and hygiene risk factors associated with the presence of feline panleukopenia in uni- and multivariate analyses.

Variables	Category	Number of Cats with Feline Panleukopenia (*n* = 42)	Univariate Analysis	Multi-collinearity	Mulitvariate Analysis AIC ^3^ Model		
			*p* ^1^	VIF ^2^	*p*	OR ^4^	95% CI ^5^
age group	young (≤2y ^6^)	40/42	**<0.001**	*1.0*	0.008	71.8	2.9–1772.7
	adult (>2y)	2/42			Ref. ^7^	Ref.	Ref.
FPV ^8^ antibody titre	titer ≥ 10	30/39	**0.022**	*1.0*			
	titer < 10	9/39					
application of hyperimmune serum in outbreak situation	yes	30/42	**<0.001**	*1.3*			
	no	12/42					
vaccination	yes	2/42	**<0.001**	*3.9*	Ref.	Ref.	Ref.
	no	40/42			**<0.001**	46.5	11.7–184.9
vaccine brand used	PureVax RCP^®^	0/2	0.734				
	Nobivac^®^ RCP	1/2					
	Virbagen^®^ felis RCP	1/2					
	Versifel^®^ CVR	0/2					
correct immunisation series *	yes	2/42	**<0.001**	*3.7*			
	no	40/42					
housing	single-housed	10/42	0.410				
	group-housed	32/42					
litterbox cleaning	1 per d ^9^	5/42	0.641				
	2 per d	37/42					
litterbox disinfection	weekly	34/42	**0.029**	20.7			
	as required	8/42					
documentation of medical history	yes	5/42	**<0.001**	12.6			
	no	37/42					
routine quarantine in d	0	29/42	**<0.001**	233.7			
	7	8/42					
	14	5/42					
disinfectant brand	Virkon^®^ S	29/42	**<0.001**	41.1			
	Bowi-Sept^®^	13/42					
	VENNO^®^ Vet 1	0/42					
disinfectants’ effectiveness against non-enveloped viruses	yes	29/42	**0.020**	19.7			
	no	13/42					
general use of hyperimmune serum in the shelters	yes	29/42	**0.020**	19.7			
	no	13/42					
dishwasher use	yes	5/42	**<0.001**	12.6			
	no	37/42					
protective clothing	yes	34/42	**0.030**	20.7			
	no	8/42					
separated isolation area	yes	34/42	**0.030**	20.7			
	no	8/42					
footbath in isolation area	yes	29/42	**0.020**	19.7			
	no	13/42					
employment ** of staff members	permanent employment	34/42	**0.030**	20.7			
	partial employment	8/42					

Factors that proved to be significant in univariate analysis (*p* ≤ 0.05) became part of the multivariate analysis, in which they were examined for multicollinearity via variance inflation factor (VIF). Variables with a VIF ≥ 5 suggested multicollinearity and were excluded from further analyses. Variables with a VIF < 5 were considered as not being multicollinear and subsequently used for further stepwise backward elimination analysis based on the Akaike information criterion (AIC). Bold values indicate statistical significance; italics values indicate multicollinearity. Values in blank table cells were eliminated either after univariate analysis (*p* > 0.05) or by stepwise backward elimination. ^1^ *p*, *p* value; ^2^ VIF, variance inflation factor; ^3^ AIC, Akaike information criterion; ^4^ OR, odds ratio; ^5^ CI, confidence interval; ^6^ y, years; ^7^ Ref, reference value; ^8^ FPV, feline panleukopenia virus; ^9^ d, days; * correct immunization series according to current vaccination guidelines [25,26]; ** employment with different salary (permanent employment with a salary of >€450.0 per month; partial employment with a salary of <€450.0 per month).

**Table 3:** Medical, husbandry, and hygiene risk factors associated with presence of fecal parvovirus shedding as detected by qPCR in uni- and multivariate analysis.

Variables	Category	Number of Cats with Fecal Parvovirus Shedding (*n* = 73)	Univariate Analysis	Multi-collinearity	Mulitvariate Analysis AIC ^3^ Model		
			*p* ^1^	VIF ^2^	*p*	OR ^4^	95% CI ^5^
age group	young (≤2 y ^6^)	70/73	**<0.001**	*1.0*	**<0.001**	10.9	2.8–42.2
	adult (>2 y)	3/73			Ref. ^7^	Ref.	Ref.
FPV ^8^ antibody titre	titer ≥ 10	57/69	**0.037**	*1.0*			
	titer < 10	12/69					
application of hyperimmune serum in outbreak situation	yes	29/73	**0.015**	*1.3*			
	no	44/73					
vaccination	yes	39/73	0.773				
	no	34/73					
vaccine brand used	PureVax RCP^®^	15/39	NA ^9^	NA	NA		
	Nobivac^®^ RCP	13/39					
	Virbagen^®^ felis RCP	6/39					
	Versifel^®^ CVR	5/39					
correct immunisation series *	yes	36/73	0.413				
	no	37/73					
presence of feline panleuko-penia	yes	29/73	**<0.001**	*1.3*	**0.033**	2.3	1.1–5.0
	no	44/73			Ref.	Ref.	Ref.
housing	single-housed	14/73	**0.013**	*1.1*	Ref.	Ref.	Ref.
	group-housed	59/73			**0.025**	2.4	1.2–5.6
litterbox cleaning	1 per d ^9^	7/73	0.205				
	2 per d	66/73					
litterbox disinfection	weekly	44/73	**0.015**	34.4			
	as required	29/73					
documentation of medical history	yes	23/73	**0.031**	30.1			
	no	50/73					
routine quarantine in d	0	21/73	**0.030**	730.2			
	7	29/73					
	14	23/73					
disinfectant brand	Virkon^®^ S	21/73	0.262				
	Bowi-Sept^®^	36/73					
	VENNO^®^ Vet 1	16/73					
disinfectants’ effectiveness against non-enveloped viruses	yes	37/73	0.161				
	no	36/73					
general use of hyperimmune serum in the shelters	yes	37/73	0.161				
	no	36/73					
dishwasher use	yes	23/73	**0.031**	30.1			
	no	50/73					
protective clothing	yes	44/73	**0.015**	34.4			
	no	29/73					
separated isolation area	yes	44/73	**0.015**	34.4			
	no	29/73					
footbath in isolation area	yes	37/73	0.161				
	no	36/73					
employment ** of staff members	permanent employment	44/73	**0.015**	34.4			
	partial employment	29/73					

Factors that proved to be significant in univariate analysis (*p* ≤ 0.05) became part of the multivariate analysis, in which they were examined for multicollinearity via variance inflation factor (VIF). Variables with a VIF ≥ 5 suggested multicollinearity and were excluded from further analyses. Variables with a VIF < 5 were considered as not being multicollinear and subsequently used for further stepwise backward elimination analysis based on the Akaike information criterion (AIC). Bold values indicate statistical significance; italics values indicate multicollinearity. Values in blank table cells were eliminated either after univariate analysis (*p* > 0.05) or by stepwise backward elimination. ^1^ *p*, *p* value; ^2^ VIF, variance inflation factor; ^3^ AIC, Akaike information criterion; ^4^ OR, odds ratio; ^5^ CI, confidence interval; ^6^ y, years; ^7^ Ref, reference value; ^8^ FPV, feline panleukopenia virus; ^9^ NA, not applicable; * correct immunization series according to current vaccination guidelines [25,26]; ** employment with different salary (permanent employment with a salary of >€450.0 per month; partial employment with a salary of <€450.0 per month).

## Data Availability

The authors confirm that the datasets analyzed during the study are available from the corresponding author upon reasonable request.
